# Users involvement in the electronic health information systems development process in Uganda: what is missing in relation to requirements gathering and analysis

**DOI:** 10.1093/oodh/oqae020

**Published:** 2024-06-25

**Authors:** Christine Kalumera Akello, Josephine Nabukenya

**Affiliations:** Department of Computer Science, Gulu University, Gulu, Uganda; Department of Information Systems, School of Computing and Informatics Technology, Makerere University, Kampala, Uganda

**Keywords:** user involvement, electronic health information systems, requirements gathering, systems analysis and design, digital health, usability

## Abstract

User involvement in the electronic health information systems (eHIS) development process is crucial for gathering and analysing requirements that accurately reflect user needs. This is because their involvement is linked to the gathering and analysis of requirements that align with user needs. However, several studies reveal that there is still limited user involvement during these crucial phases, leading to the development of ineffective and inefficient systems that do not reflect user needs. Thus, this study explored how users were involved in the requirements gathering and analysis phases during eHIS development, with an aim of identifying the missing elements that hindered the design of more effective and effective eHIS. A cross-sectional survey, encompassing secondary and primary users, explored their involvement in the requirements gathering, analysis and design phases, using both open-ended and close ended questionnaires. Respondents (*n* = 140) were purposively selected from 20 organizations in northern and central Uganda. Data were cleaned and analysed using Microsoft Excel. The findings revealed a dominant use of a top-down approach, favouring the capture of high-level requirements at the Ministry of Health level, and among implementing partners. However, less attention was given to gathering and analysing requirements from facility-level users. Even when collected, primary users reported that their opinions and recommendations were often ignored/disregarded, resulting in eHIS designs with usability-related challenges. This study underscores the critical need for active user involvement in the early stages of eHIS development to ensure alignment with user needs and work practices.

## INTRODUCTION

Recently, the Ministry of Health Uganda (MoH) adopted several Electronic Health Information Systems (eHIS), with the support of donors and development partners, to enhance its citizens' healthcare and public health delivery through control, prevention, and survey of diseases [1]. MoH Uganda recognizes eHIS as a key enabler for supporting health systems to deliver good health to its citizens [2]. Particularly, MoH uses its eHealth strategy to provide principles that guide and underpin the planning, and eHIS Implementation, to ensure the effectiveness and sustainability of eHIS in Uganda [3]. These guidelines focus on local partners' usability and involvement in developing and supporting information systems. In addition, the eHealth strategy guideline *4.6 (e),* emphasizes ‘stakeholders' active engagement in designing and delivering eHIS solutions at the local and national levels for all public, private, and partner organizations’. According to [4], ‘eHIS’ refer to a computer-managed health information system designed to improve healthcare quality, efficiency, diagnosis, treatment and patient safety; it includes mHealth, eHealth, Telemedicine, and Telehealth systems. Examples of eHIS used in Ugandan health facilities include; the Integrated Disease Surveillance and Response System and Open Medical Records Systems (OpenMRS) [5], District Health Information System version 2 (DHIS2) [6], and Mobile Tracking for Routine Weekly Surveillance Report (MTRAC) [7].

Despite Uganda's MoH committing to user involvement in eHIS development, a closer look reveals limited and problematic understanding of this concept. The currently employed top-down approach is heavily reliant on donor agencies and technical experts; which marginalizes system users at the facility level, silencing their invaluable perspectives on healthcare system needs and workflow realities [8]. While user acceptance testing takes center stage, opportunities for meaningful participation in earlier, crucial phases like requirements gathering, analysis, and design remain restricted. For instance, in the pilot studies, most systems were evaluated for efficiency and effectiveness [9–11]. In scenarios where the development team included new modules in the adopted systems, it was unclear what the users contributed to such initiatives [12]. In co-designing, users were involved from the initial stages and throughout the development process [8, 13]. Therefore, most eHIS were developed for users without their direct involvement, leading to a disregard for the local context [8]. This limited user involvement in requirements gathering and analysis, amplifies pre-existing power dynamics within the health system, where voices from the ground struggle to be heard. Such limited involvement contributes to a range of unintended consequences, including user interface complexity, data inaccuracy and incomplete information arising from poorly integrated and dysfunctional systems [3].

The aforementioned challenges make it difficult for users to recognize the value these systems add to their routine workflows [8]; and inhibit the country from reaching the sustainable development goal 3 (3.d). This is because it weakens a country's capacity to efficiently assess and monitor the health sector if eHIS data is incomplete, inaccurate or untimely [14]. Consequently, most of these systems have either stalled or stopped operations with the stoppage of donor funding [13], lack local ownership [3], have proven to be nothing more than proof-of-concepts demonstrated in a limited context, and or lack sustainability [1]. For instance, in 2012, the Ugandan government imposed a moratorium on pilot eHIS; because the public mistrust in these systems innovations had heightened [15].

Several scholars advocate for active user involvement in the system development process to address the aforementioned challenges. This is because such involvement leads to the design of efficient and effective systems, which offer context-based solutions aligned with user needs [16–18], and workflows [13, 19] and foster ownership [20]. In the information systems field, ‘User involvement’ refers to users' participation during the system development process [21]. It is primarily related to the user's perception of the system's usefulness, which fosters a sense of ownership and a more positive attitude toward computer systems [22]. There are three categories of users: primary users (regularly use the system), secondary users (occasionally use the system) and tertiary users (beneficiaries of the system) [23].

Based on the preceding discussion, it is evident that user involvement remains limited during the requirements gathering and analysis phases during eHIS development. Therefore, this study was motivated by the need to explore how users were involved during these phases, with an aim to identify the missing elements that hindered designing of more effective and efficient eHIS. Uganda was chosen as the context, to highlight specific challenges and opportunities related to user involvement in eHIS development within a developing country. Understanding these specificities helped to inform strategies for more effective user involvement during eHIS development not just in Uganda but also in other developing countries.

### Overview of the notions of requirements gathering, analysis, and design phases

‘Requirements gathering’ is a phase in the system development life cycle that involves understanding the system's context of use, and identifying user needs/preferences [24]. The ‘system analysis’ phase involves inspecting generated user requirements for usability [24], verifying that the identified requirements conform to the description of the system, are complete, consistent, not ambiguous, and do not contradict the stakeholders' expectations [25]. The ‘design’ phase involves engaging users in a controlled setting, to test the system prototype to identify bugs [24].

These three initial phases are the foundation of the software development process upon which all systems are built [26]. This is due to the fact that these phases entail identifying and interpreting user needs, in a more precise and understandable manner, and translating them into system requirements [26–28]. These phases influence the direction of the development process, system quality and costs [28–30]. When requirements are incorrectly specified or interpreted in an ambiguous, incorrect or incomplete format from the start, they lead to the development of ineffective systems that do not reflect users' needs [31], or fail to work to the expectation of the targeted user [32–34]. Furthermore, errors made during these phases are challenging to modify; this is because they require more resources (i.e. time and money) to correct [25, 33].

However, these phases are often faced with challenges such as; ignoring user needs, poor communication between users and the developers' team, which makes it challenging to articulate user needs [35, 36, 37, 38]. These challenges result in the gathering of incorrect, incomplete and inconsistent system requirements that do not reflect user needs [32, 33, 39].

### Global health institutions' perception of user involvement in eHIS development process

WHO strongly encourages using the User Centred Design (UCD) approach when designing eHIS [40]. ‘UCD’ is an interactive approach to system development that entails researching, comprehending, and considering user viewpoints in every design activity [23]. In addition to its extended benefits, UCD approach follows global digital development principles [41] that urge practitioners to iteratively design with the users and be collaborative [42]. The principles of digital development have also been endorsed by several global health institutions, including the Bill and Melinda Gates Foundation, United Nations agencies and the United States Agency for International Development [43] among others. Although research studies, white papers, and news articles offer guidelines on how to conduct UCD for global health [42, 44]; there is still concern that this approach may just be used theoretically in global health, and not done practically in the design of eHIS [45–47].

Therefore, several global and development organizations are exploring how they might design eHIS tools using the UCD approach, to address the contextual complexities of delivering health care in challenging settings [42]. However, few resources provide rigorous academic literature for further reading that system developers can use to guide them through the requirements gathering, analysis and design phases of the eHIS development process [43].

### Evidence of user involvement during eHIS development process in Uganda

Table 1 shows the description of eHIS used in Uganda health facilities and the extent to which users were involved during their development process.

**Table 1:** Description of eHIS examples, and the extent to which users were involved during their development process

**Digital health system**	**Description**	**Extent of user involvement in the eHIS development process**
MTRAC (7)	It provides managers at the district and national levels with timely surveillance data, while crowdsourcing service delivery and complaints from anonymous hotlines to improve response and accountability [7]. Its electronic monitoring initiative includes components that advocate for stakeholder involvement in reporting service delivery bottlenecks in order to generate dialogue and action where failure occurs [7]	The first evidence of user involvement in MTRAC, was during the user acceptance testing at a data users conference organized to identify any challenges in the system [7], where users generated numerous requirements from the errors they identified [48] During the upgrade of MTRAC to MTRAC pro, users provided their opinion of MTRAC Pro's learnability, satisfaction, and usefulness in weekly surveillance reporting, through questionnaires that were administered to them [48].
Uganda EMR/ OpenMRS	It is an open-source system that monitors HIV patient data and harmonises medical records systems [49]	Users were only involved in training on how to file, index, store and track data, as well as train on how to use, install, and set up the system and clear backlogs before and after it was rolled out [49].
DHIS2	It is a free and open-source software platform for creating, storing, and sharing surveillance reports [50, 51]. Some DHIS2 installations in Uganda include; the National HIS instance [52], eMTCT SMS reporting instance for prevention of HIV/AIDS from mother to child with Option B+ programme [50] It facilitates case investigation and laboratory linkage for notifiable diseases [53].	The Ugandan team was trained on how to use the system as part of user involvement [12] They further participated in developing modules within the DHIS2, like a mobile reporting module and a maternal and child health tracking module [12].
U-report	It allows Ugandan youth to send free text messages to respond to polls, receive information, and express their views on issues important to them, such as social policy, child protection, and health [7, 54].	UNICEF-Uganda created a web platform and software application to manage communication between social communicators and central managers. Users were only involved during the piloting phase [7].
Smarthealth app	It is an open-source mobile platform that supports disease diagnosis, improved performance management, real-time monitoring of diseases, and delivers health education on health behaviour by short message service, smart workflows for pregnancy care, management of childhood diseases, nutrition and immunization tracking [11]	According to [55] user perspectives were considered throughout the development process, validation of workflows and user acceptability testing to fix bugs. Users were also trained and supported to ensure product viability, utilization, quality, efficiency and effectiveness.
Stre@mline	It is an eHealth platform that enables clinicians in low-resource settings to deliver patient healthcare efficiently. It incorporates consultation, prescriptions, insurance, patient investigation, monitoring drug shortage, stockout and expiry, monthly reporting and patient follow-up on over 60 000 patients on long-term treatment [13]	In terms of user involvement, the Physicians initiated and drove the system development process, ensuring that all necessary requirements were captured. [8]. The technical design was made by istreams, while Kisiizi Hospital did the clinical design [8]. Continuous and consistent user involvement led to the generation and analysis of requirements that reflected user needs and the development of an efficient and effective system [13].
Surgical quality assurance database (SQUAD) in Uganda	The SQUAD is used to facilitate understanding the disease burdens and outcomes [56]	It was iteratively developed and refined by the SQUAD development team, which comprised; Anaesthesiologists, Data Clerks, Biostatisticians, Project Data Management Coordinator, Psychiatry Nurses, Midwife-Nurses, Data Entrants, and a systems developer [57]. In addition, formal and informal discussions were held with users to generate system requirements, analyse and co-design the prototype [57].
Rapid short message service (Rapid SMS)	It is an open-source framework for rapid prototyping and development of software applications that use SMS or text messaging [7].	The framework was created by programmers who worked directly with users on actual project implementation to ensure that the software meets their needs [7].
Clinic Master	It is an integrated billing software that monitors pharmacy and consumables inventory, accounts receivable, cash or insurance, patient visits, appointments, medical conditions, and medical history; the system is customized to use the existing MoH HMIS tools [58].	Users were only involved during the piloting of the system [58].

### Implementation challenges resulting from limited user involvement in eHIS development process

Table 2 shows challenges users experienced when the development team did not fully involve them during the eHIS development process of systems used in Uganda health facilities.

**Table 2:** Implementation challenges experienced by users when not fully involved during eHIS development process

**eHIS**	**Implementation challenges due to limited user involvement during eHIS development process**
OpenMRS	Unfriendly user interface and incomplete data due to information gaps [59].
DHIS	DHIS2 forms are not consistent with users HMIS registers [60]. The DHIS2 SMS functionality does not cover the ability to send feedback, on how specific indicators are performing at the aggregate level, although it is vital for evaluation [7]. Users experience inconsistencies between the HMIS data tools and their representations in DHIS2; the annual reports are inconsistent with the hardcopy, contain system errors, incomplete data, limited functionality and missing data quality checks [48]
MTRAC	The syntax for the feedback on sending a report is inconsistent with the information forwarded; users obtain fewer feedback messages than report messages sent, ambiguous feedback when a reporter sends a report, the system has missing fields on the forms [48]. Users experience challenges with extracting data from the MTRAC dashboard [7]; structural issues with the HMIS weekly surveillance form, since it did not disaggregate data for items by type on the forms [61]
U-report	Poll queries were challenging to understand, because system developers did not involve them in formulating system questions. Besides, when Patrons captured children's views and submitted them to the application on their behalf, it was not easy to distinguish the children's voices from the database [7]. The system does not allow anonymous communication; this limits user involvement in providing their views. Some users, especially in rural areas, found it challenging to understand the poll questions due to the language barrier [7].
Stre@mline system	The system lacks data portability between hospitals since it can only store data in local networks [13].

## MATERIALS AND METHODS

A cross-sectional survey was conducted to investigate how requirements gathering and analysis tasks were carried out, identify who was involved in these tasks, ascertain the challenges encountered, and what was missing in relation to these phases. This study design was employed because the researchers wanted to understand in-depth the current state of stakeholder involvement practices in the requirements gathering and analysis phases.

The study received approval from the Gulu University Institutional Review Board under reference number GUREC-2021-73, and also obtained permission from the Uganda National Council of Science and Technology; and secured administrative clearance from the District Health Officers of the chosen districts. Informed consent was sought from the selected respondents, who were provided with details about the study's purpose and asked to sign a consent form if they expressed willingness to participate.

**Inclusion criteria: –** this encompassed respondents’ healthcare workers who frequently used the eHIS to perform their job duties**,** herein referred to as "primary users," and the development team members involved in the design of the eHIS, herein referred to as "secondary users”.

**Exclusion criteria**: patients and patient care takers herein referred to as ‘tertiary users’ were excluded from this study.

**Study design –** Both closed and open-ended questionnaires were utilized, as depicted in the annexes located in the supplementary materials. Section A contained the background information on the respondents' organization (nature of organization, location, size and respondents' title). Section B contained questions that examined how the requirements gathering and analysis tasks were conducted, who was involved in these tasks when developing eHIS, how they were involved, and the challenges they encountered during requirements gathering and analysis tasks.

**Data collection** – quantitative data were gathered to acquire a more comprehensive and nuanced understanding of the phenomenon under investigation. The quantitative aspects of the study involved conducting a survey where data was collected using two distinct instruments; electronic questionnaires administered via email, and paper-based questionnaires administered in person. A 90% response rate was achieved.

**Data analysis and interpretation –** Responses from close-ended questionnaires were entered into an MS Excel spreadsheet, with each row representing a respondent and each column representing a question. The data was cleaned by removing irrelevant or incomplete responses, correcting formatting errors, and ensuring consistency. Numerical codes ('1′ for 'yes' and '0′ for 'no') were assigned. Descriptive statistics for each question were calculated using Excel functions to count the number of responses per option. Bar and pie charts were employed to provide a visual summary of the responses, facilitating interpretation. We also examined the open-ended responses to understand their content and identify any recurring patterns. The responses were manually coded to represent the theme. The following themes emerged from the thematic analysis; how requirements gathering and analysis tasks were conducted, extent of user involvement, and the challenges encountered during eHIS development process. Thereafter, we counted the number of responses that fell under each theme through tallying and represented them in percentages. Finally, the data were analysed and explanations provided.

## RESULTS

A total of 36 health-practitioners were purposively selected from 13 key health institutions located in the Northern and Central regions of the country, that is, Gulu and Kampala, respectively. The limited number of health institutions using EHIS in Uganda, coupled with time and cost constraints informed the rationale for purposive sampling. The roles of the respondents ranged from biostatistician, counsellors, data clerks, health information systems specialist, lab technicians, clinicians, nurses, quality control officers, to monitoring and evaluation officers.

### Distribution of respondents by study district

The distribution of primary user respondents per district was as follows; majority were from Lira (40.1%), followed by Gulu (30.5%) and Luwero (14.4%). The remaining districts had a much smaller proportion of primary user respondents, i.e. Kampala (9%), Dokolo (2.0%), Alebtong (2.0%), Oyam (2.0%) and other districts (i.e. Omoro and Nwoya) (2.0%). For secondary user respondents, majority were from Kampala (77.7%), while (22.3%) were from Gulu.

### Distribution of respondents by health facility

Among the primary user respondents, those from government hospitals, private hospitals, health centers III and IV, and other organizations affiliated with health comprised (39.2%, 33.3%, 21.6% and 6.9%), respectively. For secondary users, Hagvan Technologies, HIISP Uganda, Baylor Uganda, Infectious Diseases Institute, Ministry of Health, and the Monitoring and Evaluation Technical Support accounted for (5%, 30%, 20%, 10%, 5% and 28%) of the respondents, respectively.

### Representation of respondents by size of the organization

**Figure 1:**
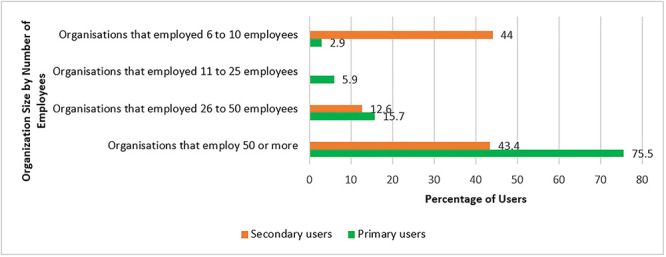
Representation of respondents by organization size.

In terms of the organization’s size, most primary user respondents (75.5%) were from health facilities employing 50 or more employees, while (15.7%) were from facilities with 26 to 50 employees. Even fewer primary user respondents (5.9%), were from facilities with 11 to 25 employees. The smallest percentage (2.9%) of primary user respondents was from facilities with 6 to 10 employees. Among the secondary users surveyed (43.4%) were from organizations with 50 or more employees, (12.6%) were from organizations with 26 to 50 employees, and (44%) were from organizations with 6 to 10 employees as shown in Fig. 1. Organizations that employed 50 or more employees had dedicated IT staff, focused more on user engagement, leading to more intentional efforts to involve users, gather their needs, and incorporate their feedback. They also had a dedicated budget for user engagement activities, like workshops; while organizations those 6 to 11 employees had limited access to resources dedicated to user involvement in eHIS development process.

### Position(s) of respondents in the organization

Majority (55%) of the respondents were Data Clerks, followed by those from other professions (30%) (i.e. OVC Focal person, Records Officers, Biostatisticians), Information Assistants (10%) and medical officers (5%). Although the Data Clerks were the highest number of respondents in the primary user category, the medical officers were underrepresented.

**Figure 2:**
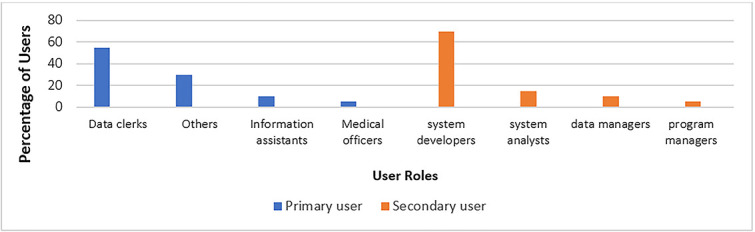
Position(s) of the respondents in the organization.

Among the secondary user respondents, majority (70%) were system developers, followed by System Analysts (15%), Data Managers (10%) and Program Managers (5%), as indicated in Fig. 2. These findings suggest that there is a diversity of roles involved in the requirements gathering and analysis tasks during eHIS development process. This is crucial for understanding the different user perspectives and needs, as well as how their varying viewpoints and priorities influenced the requirements gathering and analysis phases during the eHIS development process.

### Who is involved in requirements gathering and analysis tasks

**Figure 3:**
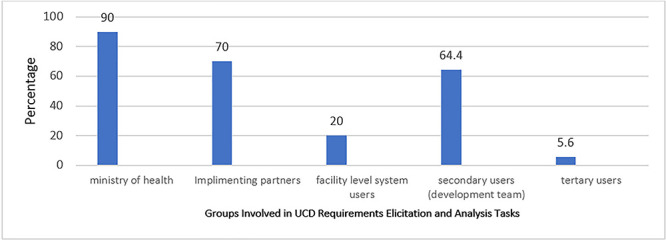
Category of users involved in requirements gathering and analysis tasks.

A substantial (90%) percentage of secondary user respondents indicated that they involved the MoH since it’s responsible for establishing requirements and standards for eHIS. A considerable number (70%) reported that they engaged with implementing partners who acted as intermediaries between developers and healthcare facilities as indicated in Fig. 3. Over half (64.4%) of Secondary users’ respondents (i.e. Program Managers, System Developers, and System Analysts), were actively engaged, since they were directly responsible for the design, development, and implementation of the system.

Facility-level system users, including; Data Clerks, Record Officers, Biostatisticians, and Monitoring and Evaluation Officers, demonstrated a slightly lower (20%) but still significant level of involvement. This low level of involvement may have been due to the fact that they were not directly involved in the development process. However, their involvement was still crucial, as they provided valuable feedback on the usability and functionality of the system. Only (5.6%) of respondents mentioned that they involved tertiary users by obtaining their feedback on the effectiveness of the eHIS. These results demonstrate that a multi-dimensional approach to user involvement was used during requirements gathering and analysis to ensure that eHIS development meets both user, technical and regulatory requirements, ultimately benefiting the healthcare sector.

### How requirements gathering and analysis tasks were conducted

**i. Responses from the secondary users**

**Figure 4:**
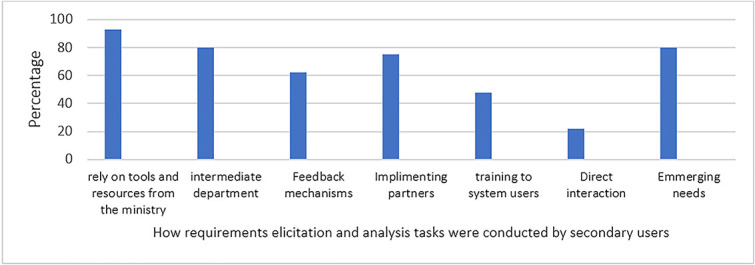
How requirements gathering and analysis tasks were conducted by secondary users.

A larger majority (93%) of secondary user respondents reported that they relied on tools and resources provided by the MoH as indicated in Fig. 4. This indicates that MoH played a central role in providing the necessary frameworks and guidelines for system development. The heavy reliance on pre-defined tools and resources from the MoH suggests a less iterative approach used in requirements gathering and analysis. For instance; while the top-down approach in requirements gathering offers structure and efficiency, it risks overlooking user needs due to limited interaction with users at the facility level. This can lead to misunderstandings, misinterpretations, and ultimately, the development of systems that do not reflect user needs.

Majority (80%) of the respondents engaged with intermediate departments that interface with high-level officials, such as the Minister and Permanent Secretary in the MoH. This suggests that by acting as conduits for communication with high-level decision-makers, these departments facilitated alignment between eHIS development and broader healthcare goals through information sharing, feedback loops and discussions, ensuring alignment between different groups.

Over half (62%) of the organizations established feedback mechanisms that system users employed to provide continuous input. This mechanism allowed for ongoing feedback, which was essential for iterative system improvement and adaptation. A significant (75%) percentage of respondents interacted with implementing partners who represented the users. These implementing partners bridged the gap between developers and users in healthcare facilities, ensuring a more seamless implementation process. A small percentage (48%) of respondents provided training to system users at healthcare facilities. This training familiarized users with the systems and served as a feedback-gathering opportunity. A smaller proportion (22%) engaged in direct interaction with users at healthcare facilities to understand their specific needs and challenges. A substantial percentage (80%) of respondents mentioned that they responded to emerging needs from stakeholders at the national level. This adaptability was important in ensuring that the electronic Health information systems (eHIS) remain relevant and effective in a dynamic healthcare environment.

**ii. Responses from the primary users.**

**Figure 5:**
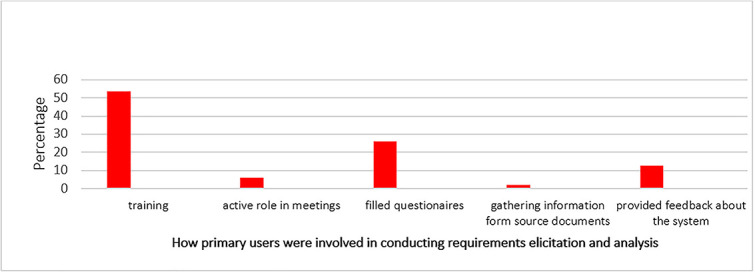
How the primary users were involved in requirements gathering and analysis tasks.

Primary user respondents (53.4%) indicated that they actively participated in training sessions as depicted in Fig. 5. These statistics suggest that trainings were prioritized as a means to ensure effective system usage and to validate its functionality. A small proportion (26%) of the respondents indicated that they made contributions by filling baseline survey questionnaires. This method, while known for its structured approach, it allowed for systematic and organized data collection in our study.

A very small proportion (12.7%) of the respondents acknowledged that they provided feedback about the system, emphasizing the invaluable role of feedback in system improvement and refinement. This response indicates that users actively participated in the requirements gathering and analysis phases by collaborating with a team of developers. Their involvement encompassed providing feedback, identifying areas for improvement, specifying system requirements, and receiving training related to system administration. This user involvement was essential for ensuring that the resulting system aligns with the needs and expectations of its intended users.

An insignificant percentage (5.9%) of respondents reported active participation in meetings dedicated to system discussion and improvement recommendations, organized by implementing partners. These central meetings fostered vibrant discussions and ideas exchange, generating valuable insights, demonstrating active user participation in collaborative tasks during requirements gathering and analysis. However, while the commitment from implementing partners was evident, unresolved issues highlight the need for effective follow-through and communication during implementation.

An insignificant proportion (2%) of the respondents mentioned that they were involved in the process of gathering information from source documents, highlighting the relative clarity of this approach when compared to others. In this method, participants diligently collected information and source documents from all departments within the facility. The low score for this activity may have been due to limited access to documents, permission restrictions to the documents, and lack of training on how to effectively identify and collect relevant documents.

### Extent of user involvement in requirements gathering and analysis tasks during eHIS development process

When asked about the extent of user involvement during eHIS development process, the secondary user respondents provided the following insights; a significant majority (70%) stated that they actively involved system users in the requirements gathering and analysis process, which reflects a strong commitment to understanding the needs and preferences of users. However, a notable minority (30%) mentioned that they did not include system users in these tasks, indicating that they opted for alternative methods or relied on intermediaries or stakeholders to gather requirements. Additionally, understanding the reasons behind the minority (30%), who did not involve system users in the requirements gathering and analysis phase, could provide insights into their practices and potential areas for improvement. On the contrary, a significant portion of primary users (47%) reported that they were never involved in eHIS development, which raises questions about the depth of their understanding and input in the development process.

**Figure 6:**
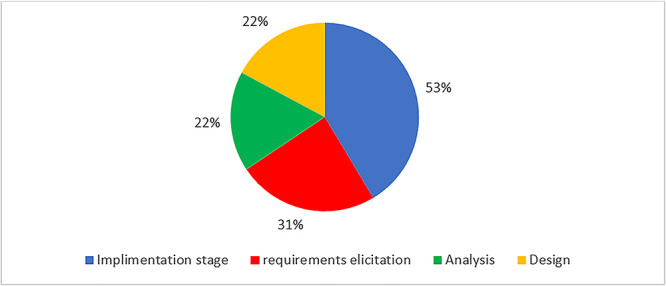
Extent of user involvement in eHIS development process.

When asked about their level of involvement throughout the various stages of the eHIS, majority (53%) of the primary users primarily focused on the later stages were actively participating in the implementation phase. In contrast, fewer (31%) primary users were involved in the earlier stages of requirements gathering, analysis, and design, i.e. only taking part in the initial requirements gathering phase and a smaller percentage (22%) in the analysis and (22%) design phases respectively, as shown in Fig. 6. These findings suggest a potential disconnect or imbalance between involving system users in requirements gathering and their level of involvement in the broader development process. This analysis indicates an opportunity for organizations to enhance user involvement particularly during requirements gathering and analysis, to ensure that the requirements gathered reflect user needs.

### Challenges encountered when executing requirements gathering and analysis tasks during eHIS development process

When asked to provide challenges encountered when executing requirements gathering and analysis tasks during eHIS development, both the secondary and primary users provided challenges that exhibit common themes, along with distinct issues specific to each group, as discussed below;

**a) Common challenges encountered by both secondary and primary users**

Misinterpretation of user needs was a challenge reported by both secondary users (70%) and primary users (56%). Secondary users encountered challenges in comprehending and interpreting user needs, while primary users expressed concerns about their needs being misinterpreted. These findings shed light on the complexities and potential pitfalls inherent in the requirements gathering and analysis tasks during eHIS development process.

Limited user participation was a shared concern among both secondary (70%) and primary (44%) users. Both groups emphasized that limited user participation in these tasks resulted in incomplete, biased, or impractical requirements, which affected the quality of the eHIS e.g. usability related challenges experienced during the implementation phase.

Ineffective communication was reported by both secondary user respondents (55%) and primary user respondents (67%) as a challenge that significantly impacted requirements gathering and analysis tasks. Ineffective communication is a double barrier because it hinders users from clearly expressing their requirements; while at the same time, it prevents designers from fully understanding user needs**.** This lack of mutual understanding inevitably leads to systems that miss the mark and fail to reflect the actual needs of the users.

**b) Unique challenges encountered by secondary users**

Mapping user needs to specific system requirements especially for systems that had multiple users was a significant challenge encountered by 75% of secondary users. This finding therefore, underscores the significance of UCD in the initial stages of system development. By taking time to understand the needs of all users, system designers can design efficient and effective systems that reflect user needs. Issues related to incomplete requirements, stemming from various sources including miscommunication between stakeholders and the development team, the evolving nature of project goals, or lack of understanding of user needs, were cited by 66.7% of secondary user respondents.

A large majority (66.7%) of respondents encountered challenges due to evolving requirements; while half (50%) of secondary users reported scope creep. These challenges are closely linked, indicating that secondary users often encounter shifting project scopes, which can make it challenging to keep the project on track. A partial percentage (50%) of the respondents noted lack of creativity in generating innovative solutions to address user needs and requirements; which challenge may be attributed to lack of diverse perspectives, risk-averse attitudes, or a rigid focus on existing practices.

**c) Unique challenges for primary users**

Slightly over half (54%) of the primary users’ respondents reported challenges related to conflicting requirements, while nearly half (48%) expressed concerns that their inputs were ignored during the requirements gathering and analysis process. These findings reveal the significance of addressing conflicts, achieving consensus, and ensuring meaningful stakeholder involvement during the development process to enhance the quality of eHIS development. Slightly below half (48%) of the primary user respondents mentioned that their input was frequently ignored by the development team. This neglect resulted in potential consequences, such as decreased user satisfaction and the risk of inefficiencies or system failures due to unaddressed user needs. Minority (38%) mentioned the difficulty in achieving consensus when capturing and defining user requirements.

### Limitations of the study

Notwithstanding the invaluable insights offered by the cross-sectional survey design that was used, the study was limited by drawing samples from only health facilities that were equipped with the national backbone infrastructure. As such, this restricted the generalizability of the study findings to eHIS implementations in diverse contexts.

## DISCUSSION

### Categories of users involved during UCD requirements gathering and analysis tasks

The study revealed that both secondary and primary users were involved in eHIS requirements gathering and analysis tasks.

A considerable majority (93%) of secondary user respondents mentioned that they directly involved the MoH during requirements gathering and analysis. Involving the MoH directly and interacting with high-level officials demonstrates efforts to engage key stakeholders and decision-makers; which is consistent with the collaborative approach advocated by UCD [62]. Additionally, 80% interacted with the intermediate department that interfaces with the high-level officials at the MoH. Moreover, 62% had a feedback mechanism that users utilized to provide continuous input. The existence of feedback mechanisms and interactions with implementing partners also reflects attempts to gather input from users and stakeholders; thus, promoting iterative design processes typical of UCD. Furthermore, 75% interacted with implementing partners who served as users’ representatives, such as Baylor Uganda, TASO, and IDI. Lastly, 48% provided training to system users from whom they gathered feedback. This finding resonates with existing literature, which emphasizes the importance of involving a diverse range of stakeholders to ensure that healthcare systems meet the needs of different user groups [16, 17, 23].

However, the relatively low level (22%) of interaction with users at health facilities suggests a gap in the application of UCD principles. Effective UCD entails direct engagement with users to understand their specific needs, preferences, and challenges. Without substantial involvement from these primary users, there is a risk of designing solutions that do not adequately address user requirements or workflow realities [62]. The fact that 80% of primary users responded to emerging needs from stakeholders at the national level, underscores the importance of considering diverse perspectives and contexts in UCD. This finding aligns with the core principle of UCD, which emphasizes the significance of involving a broad range of stakeholders throughout the development process to ensure that solutions meet the needs of all user groups. By incorporating input from primary users who directly interact with healthcare systems, designers can gain valuable insights that contribute to the design of more effective and user-friendly solutions [63].

While the findings demonstrate some adherence to UCD principles, such as stakeholder engagement and responsiveness to emerging needs [63], there is room for improvement regarding directly involving primary users, and ensuring their perspectives are adequately integrated into the design process. By enhancing collaboration with users at health facilities, designers can better address their unique needs and preferences, finally leading to more successful and user-centric healthcare systems.

### Level of user involvement

The findings regarding user involvement in requirements gathering and analysis reveal both adherence to, and deviations from the principles of UCD [64]. Passive methods like training sessions were predominant (53.4%), compared with active user engagement methods like questionnaires (26%), feedback (12.7%) and meetings (5.9%). Primary users expressed dissatisfaction regarding the disregard for their opinions and recommendations, which undermines user trust and participation in the development process during eHIS development process. This finding highlights the need for improved user involvement throughout the development process in line with UCD principles [62].

This study underscores the importance of user involvement during requirements gathering and analysis, as supported by [13, 16, 19, 23, 65]. However, the identified differences in working styles and communication between system developers and users highlight a common challenge in UCD; where inadequate collaboration can lead to poor understanding of user needs and eventually result in suboptimal system designs.

The revealed gap in concrete user involvement during the eHIS requirements gathering and analysis phase highlights a critical deficiency in the application of UCD principles. This deficiency is particularly pronounced among primary users, who often feel excluded from decision-making processes and struggle to influence design choices effectively. Such limited involvement can lead to disregard or underutilization of user recommendations, which contradicts the core tenets of UCD [17, 67].

The significant disparities between primary and secondary users regarding the extent of user involvement in the requirements gathering and analysis phases, exemplify the importance of inclusivity and collaboration in UCD. The identified disconnect between these user groups explains challenges encountered, such as incorrect, incomplete and ambiguous requirements; all of which undermine the effectiveness of eHIS systems [5, 7, 59].

### How are the tasks in requirements gathering and analysis conducted?

Majority (80%) of the secondary users’ respondents indicated that they engaged with intermediate departments which interfaced with high-level officials, to gather and analyse requirements that align with evolving user needs. However, this top-down approach introduces a potential challenge, i.e. the risk of hierarchy-driven biasness, and incomplete understanding of users' needs [8]. Balancing input from high-ranking stakeholders with insights from those at various operational levels becomes crucial to avoid designing systems overly focused on the needs of a select few.

A significant majority (93%) of the secondary user respondents highlighted their reliance on tools and guidance provided by MoH to determine the kind of user requirements to be gathered and analysed. While this approach aligns with Uganda’s eHealth Policy and Strategy that provide an appropriate basis for guiding the development of eHealth systems in Uganda [3]; overreliance on guidance without active user involvement may overlook specific user needs, preferences, and insights [68]. This could lead to gathering and analysis of user requirements that don’t fully align with user needs, consequently resulting in the design of eHIS with usability-related issues [5]. This finding highlights the importance of directly engaging users in the requirements gathering and analysis phases, a core tenet of UCD [67].

The most prevalent strategy used to gather and analyse eHIS requirements is the utilization of tools provided by the MoH (93%); thus, emphasizing the central role of the Ministry in influencing eHIS development. Other noteworthy strategies include interacting with intermediate departments (80%), responding to emerging user needs (80%), engaging with implementing partners (75%) and establishing feedback mechanisms (62%). In contrast, direct interaction with healthcare facility users is less prevalent (48%). This means that a significant portion of the designed eHIS requirements stem from ideas sourced either from MoH, or the implementing partners, with minimal user input at the facility level. This study therefore highlights the prevalence of the top-down approach in requirements gathering and analysis during eHIS development process. On the contrary, UCD advocates for bottom-up approaches that prioritise user involvement to ensure that the system development process is grounded in user needs and preferences [69].

Majority (75%) of the respondents mention that Implementing partners play a pivotal role in bridging the gap between the development team and primary users during requirements gathering and analysis phases. This role is consistent with the concept of intermediaries in UCD requirements gathering, that facilitate communication between different stakeholders, the market and technology [70]; whereas implementing partners involvement can facilitate communication, there's a risk of potential misinterpretation of user needs or the introduction of their own perspectives.

While domain experts within programs departments provide valuable assistance in handling technical or medical requirements, there's a risk that they may not comprehensively represent the diverse range of users' perspectives and preferences. This finding aligns with existing studies emphasizing the active participation of domain experts in the successful development of eHIS [71].

A considerable number (62%) of organizations employ an ongoing feedback mechanism that enables users to provide continuous input, suggestions, and concerns about the eHIS functionality and usability. This direct user engagement ensures that the systems requirements are continuously refined and aligned with the evolving needs and preferences of its users. The emphasis on continuous feedback mechanisms links UCD techniques with participatory design approaches [72]. Employing ongoing feedback mechanisms aligns with UCD principles by ensuring continuous refinement and alignment of the systems requirements with evolving user needs [74]. However, managing and prioritizing the influx of continuous input poses challenges such as information overload and cognitive strain**,** emphasizing the importance of efficient feedback management strategies in UCD.

### Challenges users experienced during requirements gathering and analysis

Limited user involvement during requirements gathering and analysis is identified as a challenge encountered by both secondary (70%) and primary users (44%) throughout the eHIS development process. These findings are consistent with [72, 73] where user participation is limited to a specific stage, users often end up playing the role of information providers rather than co-designers of the systems. The core principle of UCD, emphasize active participation of users in all stages of system development process to ensure that their needs and preferences are adequately addressed [63, 66].

Both primary and secondary users face significant challenges, including miscommunication (67%, and 55%), limited user participation (70% and 44%) and misinterpretation of user requirements (70% and 56%), respectively. Secondary users encounter additional difficulties, such as incorrect mapping of user needs to specific requirements (75%), incomplete requirements (67%) and lack of creativity (50%), which can lead to designing of ineffective and inefficient systems [7, 48, 59, 60]. The unique challenges encountered by primary users include; conflicting requirements (54%), difficulty in achieving consensus (38%), and ignoring their input (48%). These findings underscore the importance of thorough user engagement advocated by UCD as mitigation for such challenges [62].

The above challenges manifest into usability-related issues that compromise the overall effectiveness of the eHIS. For instance, system errors, information duplication, unfriendly user interface, system complexity, and the absence of essential system functionalities experienced by users [5, 62]. These usability-related issues further emphasize the critical role of user involvement in addressing these concerns. By actively involving primary, and secondary, throughout the development process, the development team can gain valuable insights into user needs and preferences, eventually leading to the development of more effective eHIS solutions.

## CONCLUSION

This study contributed to the understanding of users’ involvement in the requirements gathering, analysis, and design phases of eHIS development in Uganda health system context. Furthermore, the study explored what was missing in relation to these phases, which inhibited the designing of more effective and efficient eHIS. The findings showed that majority of secondary users employed a top-down approach, focusing on tools provided by the MoH, underscoring the Ministry's central role in influencing eHIS development. However, direct interaction with healthcare facility users was notably limited; which aligned with the assertion by many scholars that eHIS were often developed with minimal user involvement. Primary users predominantly relied on training sessions, supplemented by methods such as questionnaires, feedback provision, source document gathering and meetings, albeit to varying extents. Worse still, most eHIS functionalities were based on requirements from the MoH or implementing partners, rather than the needs of the health facility eHIS users. This made the eHIS susceptible to usability-related challenges during implementation e.g. system errors, unfriendly user interface, and system complexity among others.

From the findings and discussions, the study points to the need for adopting a balanced approach that actively identifies and engages all stakeholders. These stakeholders include both primary and secondary users as defined in the introduction section and described in the methodology section. Engaging such diverse stakeholders’ right from the start of the eHIS development process is crucial, since they offer valuable insights that could ensure that the system requirements that are gathered and analysed cater for their unique needs and workflows. This can ultimately lead to the design of efficient and effective systems that reflect user needs. Moreover, the study findings underscore the necessity of adhering to UCD principles by prioritizing user involvement and collaboration throughout the eHIS development process. Adherence to these principles such as active user involvement, inclusivity and iterative design, developers can mitigate the aforementioned challenges and develop eHIS that are more user-friendly, effective, and aligned to user needs.

## Supplementary Material

OOHD_Supplementary_Materials_oqae020
