# Supplementary material for: Users involvement in the electronic health information systems development process in Uganda: what is missing in relation to requirements gathering and analysis
Source: Oxf Open Digit Health. 2024 Jun 25;2:oqae020. doi: 10.1093/oodh/oqae020 (PMC11932415; doi:10.1093/oodh/oqae020)
Supplement: OOHD_Supplementary_Materials_oqae020 [file OOHD_Supplementary_Materials_oqae020.docx]

**Users Involvement in the Electronic Health Information systems Development Process in Uganda: What is missing in relation to Requirements gathering and analysis.**

Christine Kalumera Akello^a^ and Josephine Nabukenya^b^*

^a^ Department of Computer Science, Gulu University, Gulu, Uganda, https://orcid.org/0000-0001-6183-0698;

^b*^ School of Computing and Informatics Technology, Makerere University, Kampala, Uganda. https://ORCID No. 0000-0002-4731-2496. Corresponding author email: [josephine@cit.ac.ug](mailto:josephine@cit.ac.ug). Mailing address: Makerere University, School of Computing and Informatics Technology**. Plot 56, Pool Road, P.O. Box 7062, Kampala, Uganda Kampala, Uganda**

**ANNEXES: QUESTIONNAIRES USED DURING THE CROSS-SECTIONAL SURVEY**

**Annex1: SURVEY STUDY QUESTIONNAIRE: FOR DEVELOPERS OF eHIS**

**USERS INVOLVEMENT IN REQUIREMENTS GATHERING, ANALYSIS AND DESIGN TASKS DURING THE ELECTRONIC HEALTH INFORMATION SYSTEMS DEVELOPMENT PROCESS**

Introduction: This questionnaire investigates how users were involved in requirements gathering, analysis and design tasks of Electronic Health Information Systems used in health facilities in Uganda.  *Requirements gathering* is the process of obtaining your opinions and recommendations on how to improve Electronic Health Information Systems during their development process. *Analysis* and *design* involve examining the strengths and weaknesses of the current systems and processes to identify *“what”* would be required to improve the services offered. Although users’ feedback is important, in most cases, system developers ignore it during the development process.

The failure to fully involve users during requirements gathering, analysis and design tasks usually lead to the designing of electronic health information systems that users will usually abandon because they do not meet their (users) needs.

Accordingly, this study investigates the status of users’ involvement in the development process of Electronic Health Information Systems in Uganda, particularly, whether users were involved, challenges for not involving users, and recommendations for optimising users' involvement during the development process of Electronic Health Information Systems.

Given the background to and objective of this study, you have been identified as a KEY participant; because you are one of the Electronic Health Information System user(s) in your organisation. Some examples of users of Electronic Health Information Systems include; Biostatisticians, data clerks, ICT officers, patients, health workers, medical device users, and organisations with health interests or a specific disease.

Therefore, this is to request you to participate in this investigation by providing your responses in the space provided. Accepting to fill in this questionnaire is considered as your CONSENT to participate in this study. The responses to the questions will take 15 to 20 minutes.

*Please note that the findings from this study will be used for ONLY academic research purposes and will be treated as highly confidential.*

**SECTION A: ORGANISATION BACKGROUND**

1. Name of Organisation……………………………………………………………………………………….………………………..…………………..
2. Nature of organisation business……………………………………………………………………………………..……………………………....
3. Location of Organisation……………………………………………………………………………………………….…………..……………….……
4. Size of the organisation

☐☐ 2–5 employees

☐ 6–10 employees

☐ 11–25 employees

☐ 26–50 employees

☐ Above 50 employees

**SECTION B: USER EXPERIENCE**

1. Please provide your role/position in this organisation …………………………………………………………………..…………….
2. How long have you worked in this organisation?

☐ 1-5 years

☐ 6-10 years

☐ 11-15 years

☐ 16-20 years

## Do you involve users during requirements gathering and analysis when developing Electronic Health Information Systems (eHIS)?

☐ Yes

☐ No

##

## Who do you involve during in the execution of requirements gathering and analysis tasks?

……………………………………………………………………………………………………………………………………………….…………………..

…………………………………………………………………………………………………………………………………………………………………..

### How do you conduct requirements gathering and analysis tasks?

…………………………………………………………………………………………………………………………..………………………………..

……………………………………………………………………………………………………………………………………………………………..

## What challenges do you encounter when executing requirements gathering and analysis tasks during eHIS development process?

…………………………………………………………………………………………………………………………………………………………………..

……………………………………………………………………………………………………….……………………..…………………………………..

……………………………………………………………………………………………………………………………..…………………………………..

## Please provide suggestions on how to improve user involvement during the execution of requirements gathering and analysis tasks during eHIS development process

………………………………………………………………………………………………………………………………………….……………………..

………………………………………………………………………………………………………………………………………….………………………..

**Annex 2: SURVEY STUDY QUESTIONNAIRE: FOR USERS OF ELECTRONIC HEALTH INFORMATION SYSTEMS**

**USERS INVOLVEMENT IN REQUIREMENTS GATHERING, ANALYSIS AND DESIGN TASKS DURING THE ELECTRONIC HEALTH INFORMATION SYSTEMS DEVELOPMENT PROCESS**

Introduction: This questionnaire investigates how users were involved in requirements gathering, analysis and design tasks of Electronic Health Information Systems used in health facilities in Uganda.  *Requirements gathering* is the process of obtaining your opinions and recommendations on how to improve Electronic Health Information Systems during their development process. *Analysis* and *design* involve examining the strengths and weaknesses of the current systems and processes to identify *“what”* would be required to improve the services offered. Although users’ feedback is important, in most cases, system developers ignore it during the development process.

The failure to fully involve users during requirements gathering, analysis and design tasks usually lead to the designing of electronic health information systems that users will usually abandon because they do not meet their (users) needs.

Accordingly, this study investigates the status of users’ involvement in the development process of Electronic Health Information Systems in Uganda, particularly, whether users were involved, challenges for not involving users, and recommendations for optimising users' involvement during the development process of Electronic Health Information Systems.

Given the background to and objective of this study, you have been identified as a KEY participant; because you are one of the Electronic Health Information System user(s) in your organisation. Some examples of users of Electronic Health Information Systems include; Biostatisticians, data clerks, ICT officers, patients, health workers, medical device users, and organisations with health interests or a specific disease.

Therefore, this is to request you to participate in this investigation by providing your responses in the space provided. Accepting to fill in this questionnaire is considered as your CONSENT to participate in this study. The responses to the questions will take 15 to 20 minutes.

*Please note that the findings from this study will be used for ONLY academic research purposes and will be treated as highly confidential.*

**SECTION A: ORGANISATION BACKGROUND**

1. Name of Organisation……………………………………………………………………………….……………………………………………..
2. Nature of organisation business……………………………………………………………………………………………….…………....
3. Location of Organisation……………………………………………………………………………..……………………..…………….……
4. Size of the organisation

☐ 2–5 employees

☐ 6–10 employees

☐ 11–25 employees

☐ 26–50 employees

☐ Above 50 employees

**SECTION B: USER EXPERIENCE**

1. Please provide your role/position in the organisation …………………………………………………….………..…………….
2. How long have you worked in this organisation?

☐ 1-5 years

☐ 6-10 years

☐ 11-15 years

☐ 16-20 years

1. Have you ever participated when they were creating any Electronic Health Information Systems (software)?

☐ Yes

☐ No

1. How often did you participate in the creation (development process) of the Electronic Health Information Systems (*check all that apply*)

☐ Always participated

☐ Sometimes participated

☐ Rarely participated

☐ Never participated

1. At what stage did you participate when they were creating the Electronic Health Electronic Health Information Systems, you have listed above? (*check all that apply*)

☐ Requirements gathering (provide my opinion and recommendations)

☐ Analysis (studied the system strengths and weaknesses to identify what is required)

☐ System Design (showed the requirements within the business process in a diagram form)

☐ Implementation (use the system when it was rolled out in the health facilities)

☐ Monitoring and Controlling (assessed the effectiveness and challenges of the rolled-out system)

☐ Other, please specify: ……………………………………………………..………………………………………..……………………………….………

………………………………………………………………………………………………………………………………………………………………………………

……………………………………………………………………………………………………………………………………………………………..…………………

1. Please provide give examples to show how you participated in any of the stages you have ticked above (question 17) ……………………………………………………………………………………………………………………………………………….…..

……………………………………………………………………………………….……………………………………..…………………………………………

………………………………………………………………………………………………………………………..……………..………………………………………………………………………………………………………..…………………………………………………………………………..…………………

…………………………………………………………………………………………………………………………………………………..……………..……

…………………………………………………………………………………………………………………………..………………………..…………………

…………………………………………………………………………………………………………………………………………………..……………..……

1. Did you experience some problems when you participated in creating (developing) Electronic Health Information Systems (software)?

☐ Yes

☐ No

## What challenges do you encounter when executing requirements gathering and analysis tasks during eHIS development process?

…………………………………………………………………………………………………………………………………………………………………..

………………………………………………………………………………………………………………..………………………………………………..

………………………………………………………………………………………………………………..………………………………………………..

## Please provide suggestions on how to improve user involvement during the execution of requirements gathering and analysis tasks during eHIS development process

……………………………………………………………………………………………….………………..……………………………………………..

………………………………………………………………………………………………..………………..……………………………………………..

…………………………………………………………………………………………………………………..……………………………………………..
